# A Widely Used In Vitro Biofilm Assay Has Questionable Clinical Significance for Enterococcal Endocarditis

**DOI:** 10.1371/journal.pone.0107282

**Published:** 2014-09-25

**Authors:** Anne-Marie Leuck, James R. Johnson, Gary M. Dunny

**Affiliations:** 1 Department of Medicine, University of Minnesota, Minneapolis, Minnesota, United States of America; 2 VA Health Care System, Minneapolis, Minnesota, United States of America; 3 Department of Microbiology, University of Minnesota, Minneapolis, Minnesota, United States of America; University of Kansas, United States of America

## Abstract

Biofilm formation may play an important role in the pathogenesis of infections caused by *Enterococcus faecalis,* including endocarditis. Most biofilm studies use a polystyrene dish assay to quantify biofilm biomass. However, recent studies of *E. faecalis* strains in tissue and animal models suggest that polystyrene dish results need to be interpreted with caution. We evaluated 158 clinical *E. faecalis* isolates using a polystyrene dish assay and found variation in biofilm formation, with many isolates forming little biofilm even when different types of media were used. However, all tested clinical isolates were able to form biofilms on porcine heart valve explants. Dextrose-enhanced biofilm formation in the polystyrene dish assay was found in 6/12 (50%) of clinical isolates tested and may explain some, but not all of the differences between the polystyrene dish assay and the heart valve assay. These findings suggest that in studies assessing the clinical relevance of enterococcal biofilm-forming ability, ex vivo biofilm formation on a relevant tissue surface may be warranted to validate results of in vitro assays.

## Introduction

The Centers for Disease Control and Prevention and the National Institutes of Health estimate that 65–80% of all microbial infections involve biofilm formation, yet only recently have we started to understand the role of biofilms in infection [1], [2]. *Enterococcus faecalis* is a common commensal organism of the human gastrointestinal tract [3]. *E. faecalis* is capable of causing life-threatening infections such as endocarditis, and biofilm formation may play an important role in the pathogenesis of these infections [4], [5]. If so, mutations in important genetic determinants of biofilm development would be expected to reduce virulence. However, currently published data inadequately address the link between biofilms and virulence for enterococci.

Since the 1980’s, studies of biofilms have utilized an in vitro assay in which biofilms are allowed to form at the bottom of a 96-well polystyrene dish and a stain such as crystal violet or safranin is used to quantify the biofilm biomass (referred to hereafter as the “polystyrene dish assay”) [6], [7]. The majority of in vitro studies of enterococcal biofilms have used a similar assay [8], [9], [10], [11], [12]. This assay offers advantages such as ease of use and high-throughput screening of large numbers of clinical isolates or mutant libraries. Some variables in this assay, including the medium used or the plate material, can be altered, but the conditions still differ significantly from those of biofilm development during natural infections. Therefore, assessment of a given strain’s potential to form biofilms during infections on tissue, such as heart valves, based entirely on the polystyrene dish assay may have limited clinical relevance.

Two recent reports from our laboratory suggest that the significance of biofilm formation in the polystyrene dish assay needs to be interpreted with caution. One study examined the role of the enterococcal aggregation substance (AS) protein, a known virulence factor in experimental endocarditis due to *E. faecalis* [13], in early biofilm development on plastic coupons and porcine heart valve explants. It showed that AS enhanced biofilm development, and that for all isolates the process was accelerated on the valve surface relative to a plastic surface [14]. These findings suggested that colonization of the cardiac valve surface could proceed by more pathways than were operative on the plastic surface. In another recent study, isogenic strains with defined mutations that affect biofilm formation on polystyrene surfaces were compared in a rabbit endocarditis model. Although two determinants were identified that affected both in vitro biofilm formation and endocarditis virulence, several other determinants were identified for which mutation altered the in vitro biofilm phenotype but not in vivo virulence [15].

These discrepancies between in vitro biofilm phenotypes and endocarditis-causing potential suggested a need to reevaluate the biological significance of laboratory assays for biofilm formation by clinical isolates of *E. faecalis*. Such isolates are known to exhibit variability in biofilm formation in vitro and in the carriage of genes associated with virulence, such as *esp* and *asa* [16], [17]. In this study we compared the biofilm-forming ability of a large number of *E. faecalis* isolates from diverse clinical sources, and by several well-characterized laboratory strains, as measured by the polystyrene dish assay, and by a porcine heart valve explant assay. The results showed a lack of correlation between the assays.

## Methods

### Bacterial strains and growth conditions

The study isolates were selected from a large library of 402 *E. faecalis* isolates, assembled at the Minneapolis Veterans Affairs Medical Center (MVAMC) from 1994–1999. *E. faecalis* was identified using a metabolic profile index method (API-20S, bioMérieux, Durham, NC). The library consisted of consecutive extra-intestinal isolates from the MVAMC clinical microbiology laboratory and fecal isolates from hospitalized and community volunteers without gastrointestinal disease. Of these, 158 fecal, urinary, bloodstream, and wound isolates were chosen randomly and were evaluated for biofilm formation using the polystyrene dish assay. We also evaluated a well-described laboratory strain of *E. faecalis*, OG1RF [18], and 5 isogenic mutants, OG1RF *ΔahrC, OG1RF Δeep,* OG1RF Ω*argR*, OG1RF Ω*atlA*, and OG1RF Ω*pyrC*, which have been evaluated previously in both the polystyrene dish assay and an in vivo rabbit endocarditis model [15], [19]. *E. faecalis* was cultivated in brain heart infusion broth (BHI) (BD Bacto, Becton, Dickinson, and Company, Sparks, MD) under static conditions at 37°C in ambient air.

### Polystyrene dish assay and biofilm index

Polystyrene dish assays were performed according to a protocol previously published by this lab for use with *E. faecalis*, hereafter referred to as the “standard polystyrene dish assay” [15]. Overnight BHI broth cultures of each isolate were diluted 1∶100 in tryptic soy broth without dextrose (TSB without dextrose; Becton, Dickinson, and Company) and 100 uL of this mixture was dispensed into 8 wells of a 96-well flat-bottomed polystyrene dish (Costar 3595; Corning, Inc, Corning NY). Polystyrene dishes were incubated statically at 37°C in a humidified chamber for 24 hours and the optical density was read with a Modulus microplate reader (Turner Biosystems, Sunnyvale, CA) at 600 nm (OD_600_) to assess planktonic growth. The wells were washed to remove non-adherent cells and then stained with 100 uL 0.1% safranin. The wells were washed again, and the optical density of the remaining stain was read at 450 nm (OD_450_).

Since the isolates showed variable planktonic growth after 24 hours (not shown), biofilm formation was recorded as a “biofilm index”, which was obtained by dividing the OD_450_ by the OD_600_ [8]. The use of biofilm index values rather than absolute biofilm biomass adjusts for the differences in growth of planktonic cells between various strains. Biofilm index patterns were similar after 4 or 24-hour incubation (not shown). An isolate could have a negative biofilm index if its OD_450_ was less than the value for the wells containing sterile medium. Isolates with a biofilm index >1.0 were designated arbitrarily as “high-level biofilm formers”, and those with a biofilm index <0.1 as “low-level biofilm formers”. Because some other studies have evaluated biofilm formation in the polystyrene dish assay using TSB (0.25% dextrose), the assay was also completed using TSB for those strains evaluated in the heart valve assay [20]. For selected strains the assay was also performed as above but using TSB with supplemental dextrose (0.75% dextrose) and endothelial cell basal medium (EBM; 0.1% dextrose, Lonza, Walkersville, MD). For EBM, the assay was performed in both room air and at 7% CO_2_ to mimic the heart valve assay (described below) with no difference noted between atmospheric conditions (results not shown). At least 3 biological replicates were evaluated for each isolate and the results were averaged.

For quantitative culture, 4 wells for each isolate were washed as usual, then filled with 100 uL of phosphate buffered saline (PBS). The adherent biofilm was scraped free from the well using a pipette tip and the resulting suspensions were pooled, after which aliquots were removed for quantitative culture. The scraped wells were then stained with safranin and considered acceptable if no visible stain remained.

### Heart valve assay

Isolates were evaluated for early biofilm formation on tissue using an established ex vivo porcine heart valve explant model with minor variations (referred to as the “heart valve assay”) [14]. Porcine hearts were obtained from pigs that had been sacrificed in the course of other projects and had their hearts removed by Experimental Surgical Services and the College of Veterinary Medicine at the University of Minnesota. The University of Minnesota Institutional Animal Care and Use Committee determined that because the present project did not involve sacrifice of experimental animals it did not constitute research using vertebrate animals.

Once the heart was obtained, the aortic, mitral, and pulmonic valves were excised aseptically. From each excised valve, multiple 6 mm punch biopsies of valve tissue were removed and weighed. Valve pieces were then placed in EBM with 100 ug/mL gentamicin for 2–16 hours at 4°C to eliminate contaminating organisms. If valves could not be used immediately, they were frozen to −80°C in 90% fetal bovine serum and 10% DMSO. Comparison of fresh and frozen valves showed no difference in final recovery of colony forming units (CFU) per mL (data not shown). Valve sections (from the gentamicin bath or freezer) were washed three times in 4 mL PBS to remove gentamicin prior to being placed in a 6-well Costar 3516 tissue culture plate (Corning, Inc., Corning, NY). A ratio was calculated for each valve section by dividing the valve section weight by the average of all valve section weights, and this ratio was used to normalize the volumes of EBM and bacterial inocula used in assay set-up. The standard volumes were 4 mL of EBM, 10 uL of bacterial inoculum, and 500 uL of PBS (volume used for re-suspension of adherent bacteria and quantitative culture). The actual volumes used were the standard volume multiplied by the ratio.

For the biofilm assay, valve sections were placed in 4 mL EBM with 5% fetal bovine serum, 500 mg hydrocortisone, 6 mg bovine brain extract, and 0.1% human recombinant epidermal growth factor. Bacterial strains were grown statically in BHI overnight at 37°C and re-suspended in PBS at an OD_600_ of 1.5, after which 10 uL of the appropriate bacterial suspension was added to each well containing a valve segment, with subsequent incubation at 37°C in 7% CO_2_ for 4 hours, with rocking.

Valve sections were washed three times in 4 mL PBS by inverting tubes three times to remove loosely associated bacteria. They were then placed in 500 uL PBS, sonicated for 30 seconds using a VirSonic 475 probe sonicator (VirTis, Gardiner, NY), and treated with a motorized pestle (Fisher Scientific, Hanover Park, IL) for 1 minute to remove adherent biofilm bacteria. To quantify bacteria, aliquots from the buffer were plated onto Todd-Hewitt (TH) agar (Becton, Dickinson, Sparks, MD). At least 3 biological replicates were evaluated for each isolate, including OG1RF, and the valve-adherent bacterial loads (CFU/mL) were averaged. A separate valve section was inoculated with sterile EBM for each experiment to ensure that the original valve sections were sterile. Biofilm formation was considered to be present if enumerated bacteria from the valve section were present in numbers as great as or greater than those for valve sections inoculated with OG1RF. A previous analysis showed that biofilms developed during a 4-hour incubation of OG1RF with valve sections, including formation of microcolonies and production of extracellular matrix [14].

### Analysis of *asa* and *esp* presence

The laboratory strain *E. faecalis* OG1RF is *esp*-negative [21]. The MVAMC clinical isolates were evaluated for the presence of *esp* by PCR using the primers (forward) 5′ CAA ACT GCT CCT AAC GGT GTG 3′ and (reverse) 5′ GCT CCA TTA GCA CCA GTA GAC CC 3′. For confirmation of isolate 12, which appeared to express Esp protein based on ELISA (see below) but was *esp-*negative according to the above primers, primers (forward) 5′ CGA TAA AGA AGA GAG CGG AG 3′ and (reverse) 5′ GCA AAC TCT ACA TCC ACG TC 3′ were used (Invitrogen, Carlsbad, CA) [21]. The MVAMC clinical isolates were also evaluated for the presence of *asa* (the gene encoding aggregation substance) by PCR using the primers (forward) 5′ GAT TCT TCG ATT GTG TTG TAA ACG 3′ and (reverse) 5′GGT GCC ACA ATC AAA TTA GG 3′ [22].

To evaluate expression of *esp* in relation to dextrose concentration, quantitative enzyme linked immunosorbent assay (ELISA) was used as described previously, with some modifications [23]. Bacterial cultures were grown to exponential phase in both TSB without dextrose and TSB with supplemental dextrose (0.75% dextrose). Aliquots containing 10^5 ^CFU of the appropriate isolates in 50 uL of 100 mM carbonate buffer were placed in triplicate wells of a Nunc-Immuno plate (Nalge Nunc International, Penfield, NY) and incubated at 4°C overnight. Wells were washed 3 times with PBS+0.05% Tween 20 (PBST) and blocked by adding 100 uL of 2% bovine serum albumin (Sigma Aldrich, St. Louis, MO) in PBST to each well and incubating the plate at room temperature for 30 min. Wells were washed 3 times with PBST, inoculated with 100 uL rabbit antiserum [16] diluted 1∶1000 in PBST, incubated at 37°C for 4 hours, and rinsed again 3 times using PBST. They were then inoculated with 100 uL goat anti-rabbit IgG labeled with horseradish peroxidase (Invitrogen) at a 1∶3000 dilution, incubated at 37° for 2 hours, rinsed with PBST, and inoculated with 100 uL o-phenylenediamine dihydrochloride (Zymed, San Francisco, CA). As soon as yellow color was observed, the reaction was stopped by adding 10 uL of 2.5 M H_2_SO_4_ to each well. *esp* expression was quantified using the Modulus microplate reader at 450 nm. The experiment was performed in duplicate, with highly reproducible results.

### Statistical analysis

For comparisons of biofilm formation across multiple isolates, one-way ANOVA was used. For comparisons of isolate growth, biofilm formation, or *esp* expression between types of media, two-tailed t-tests were used. ANOVA and t-tests included Bonferroni corrections for multiple comparisons. For assessment of correlation, r^2^ was used. Statistical analysis was completed using Prism version 6.00, GraphPad Software, La Jolla, California.

## Results

### Clinical isolates display high variability in the standard polystyrene dish assay

**Figure 1:**
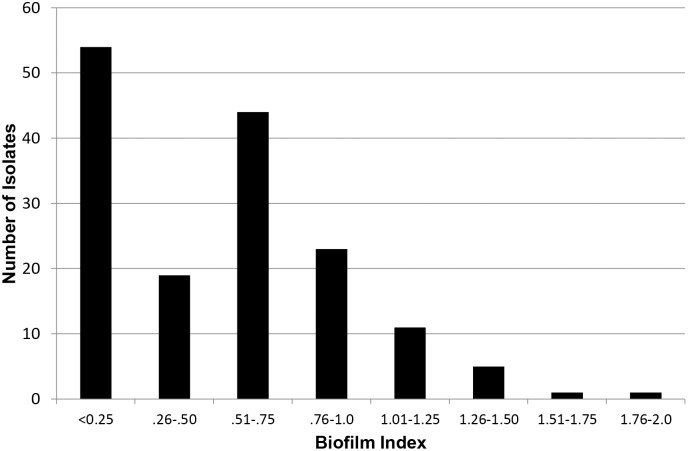
Histogram of biofilm forming ability of clinical *Enterococcus faecalis* isolates. 158 clinical *E. faecalis* isolates, including isolates from the clinical microbiology laboratory and fecal samples from healthy volunteers, were tested in the 96-well polystyrene dish biofilm assay using TSB without glucose and 24-hour incubation. To control for strain variation in planktonic growth, results are shown as each isolate’s biofilm index (Methods).

To assess between-strain variability of biofilm formation for clinical *E. faecalis* isolates in the polystyrene dish assay, biofilm index in the standard assay was calculated for each of 158 clinical *E. faecalis* isolates from sites of infection and colonization (Figure 1). A histogram of the isolates’ biofilm indices is skewed, with 117 (74%) of isolates having an index ≤0.75, and 59 (34%) having an index ≤0.25. These findings indicate that a large proportion of clinical *E. faecalis* isolates formed little or no biofilm in the standard polystyrene dish assay.

### Quantitative culture corresponds to polystyrene dish biofilm staining

**Figure 2:**
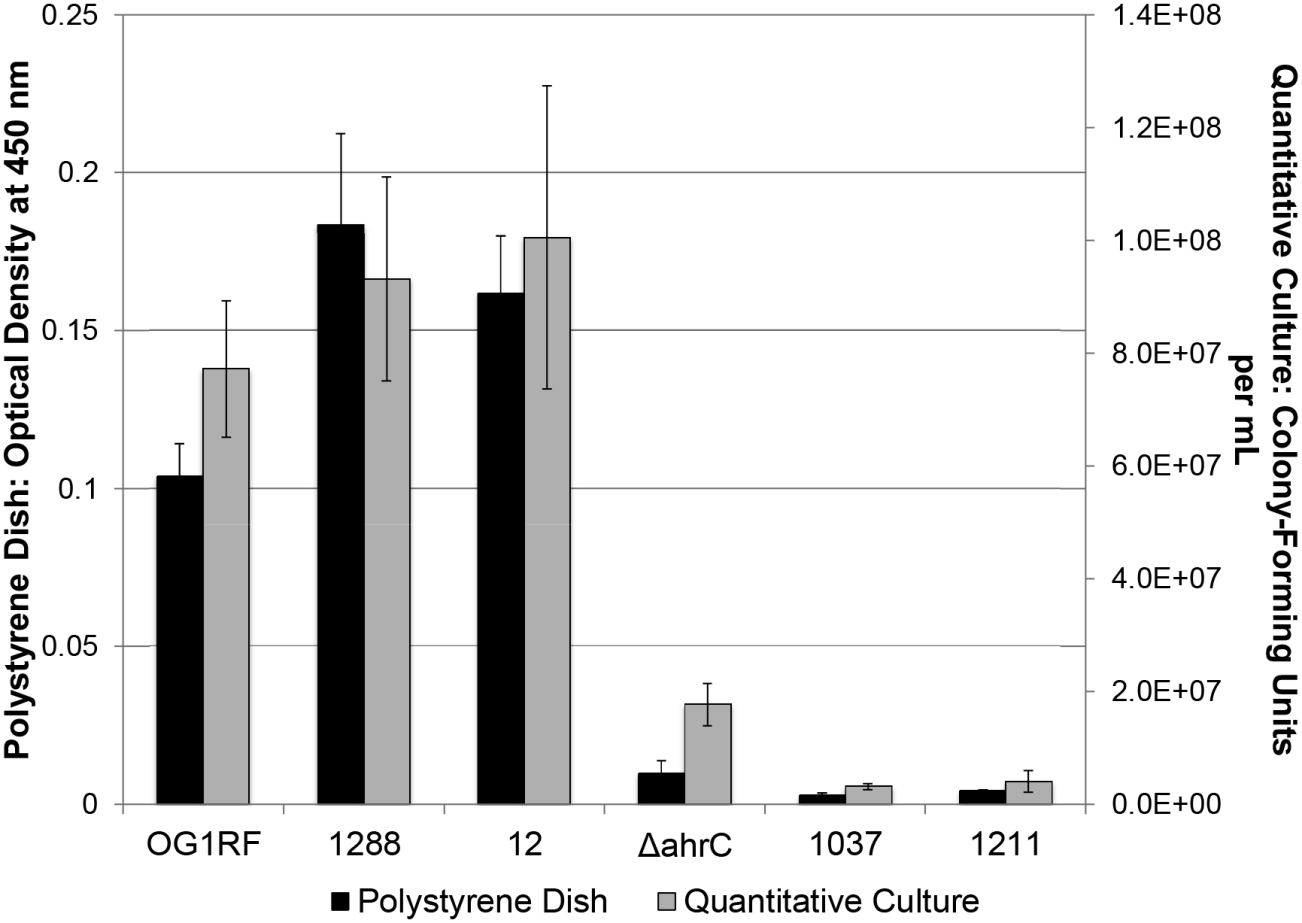
Comparison of safranin staining and quantitative colony-forming unit (CFU) counts using a polystyrene dish assay. Clinical *Enterococcus faecalis* isolates were examined for biofilm forming ability by staining for adherent biofilm biomass in a 96-well polystyrene dish biofilm assay using TSB without glucose after a 24-hour incubation. The microtiter wells were then scraped to measure CFUs present in the biofilms using quantitative culture. Error bars represent standard deviation. r^2^ = 0.95, p<0.001.

Since the biofilm biomass as measured by staining includes both bacterial cells and extracellular matrix (ECM), to separately quantify these two components the polystyrene dish wells were scraped to remove adherent biofilm cells for quantitative culture. The scraped cells were suspended in buffer, and quantitative culture results were compared to staining to clarify whether between-strain differences in biofilm biomass as observed in the polystyrene dish assay were due to differences in ECM biomass or CFU/mL (Figure 2). The safranin retention by the biofilm biomass correlated closely with the corresponding number of CFU/mL recovered from the biofilm (r^2^ = 0.95, p<0.001). These results confirmed that safranin staining of the polystyrene dish is an accurate representation of CFU adhering to the plate. However, the findings did not address the utility of this assay to evaluate the ability of bacterial isolates to form biofilms on tissue.

### The standard polystyrene dish assay is a poor surrogate for biofilm formation on tissue

To assess early biofilm formation on polystyrene vs. tissue surfaces, 6 isolates that showed high-level biofilm formation (biofilm index >1) and 6 isolates that showed low-level or no biofilm formation (biofilm index <0.1) in the standard polystyrene dish assay were compared using the heart valve assay. High-level biofilm formers were laboratory strain OG1RF and clinical isolates 12, 1172, 1234, 1278, and 1288. Low-level biofilm formers were OG1RF *ΔahrC* (a mutant of OG1RF with known attenuation of biofilm formation), and clinical isolates 99w, 103, 1037, 1150, and 1211.

**Figure 3:**
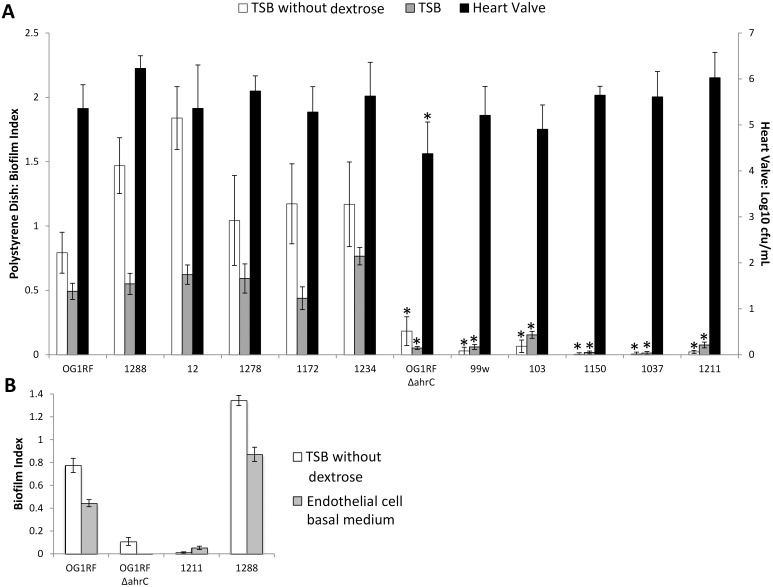
Comparison of *Enterococcus faecalis* biofilm formation using a polystyrene dish assay and an ex vivo heart valve assay. A: The laboratory strain OG1RF and clinical isolates with high-level biofilm formation, as indicated by a biofilm index >1.0 in the standard polystyrene dish assay (strains 12, 1172, 1234, 1278, and 1288), were compared to OG1RF *ΔahrC* (a mutant with attenuated biofilm formation) and clinical isolates with low-level or no biofilm formation, as indicated by a biofilm index <0.1 (isolates 99w, 103, 1037, 1150, and 1211). Isolates were compared using the polystyrene dish assay with tryptic soy broth (TSB) (0.25% dextrose) and TSB without dextrose, and using an ex vivo porcine heart valve explant assay (with medium that contains 0.1% dextrose). Polystyrene dish assays were incubated for 24 hours; heart valve assays were incubated for 4 hours. *indicates p<0.01 for less biofilm formation compared to OG1RF (for the same conditions). Note that in the heart valve assay the only strain with a significant reduction in biofilm formation compared to OG1RF is OG1RF *ΔahrC.* B: For a selection of strains representing a variety of phenotypes, biofilm formation relative to OG1RF was similar whether TSB without dextrose or endothelial cell basal medium (the medium used for the heart valve assay) was used. Error bars represent standard deviation. r^2^ = 0.98, p = 0.018.

All clinical isolates, even those that formed no biofilm in the standard polystyrene dish assay and the polystyrene dish assay using TSB (0.25% dextrose), showed biofilm formation in the heart valve model, as determined by comparing them to OG1RF (Figure 3A). For biofilm formation on heart valve explants, only negative control OG1RF *ΔahrC* differed from positive control OG1RF (mean log_10 _CFU/mL of 4.37, vs. 5.29: p = 0.004). A subset of strains showed similar results in the polystyrene dish assay with the EBM medium used in the heart valve assay (Figure 3B) (r^2^ = 0.98, p = 0.018). The attenuated biofilm formation of OG1RF *ΔahrC* across assays is supported by previously published in vivo endocarditis data that also showed attenuation of this strain compared to OG1RF [15].

### The ex vivo heart valve model correlates well with an in vivo endocarditis model

**Figure 4:**
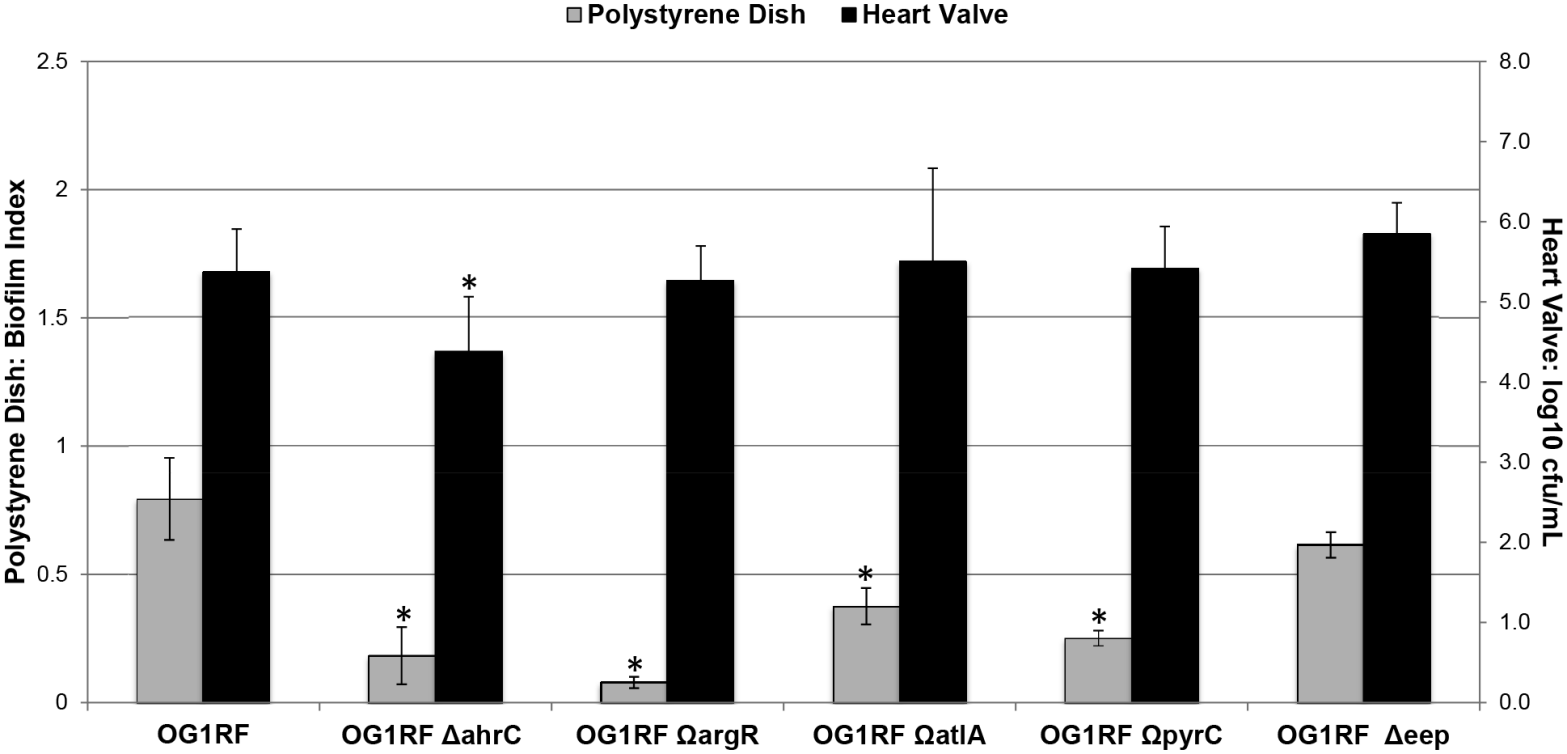
Comparison of *Enterococcus faecalis* strains previously tested in an animal model in the polystyrene dish assay vs. the heart valve assay. Strains OG1RF *ΩargR,* OG1RF *ΩatlA*, and OG1RF *ΩpyrC* were previously shown to be attenuated in a polystyrene dish assay but not in an in vivo rabbit endocarditis model. OG1RF *Δeep* has an altered biofilm phenotype resulting in no difference from the parent strain in the polystyrene dish assay but attenuation of endocarditis virulence in rabbits. These mutants, the wild type OG1RF, and OG1RF *ΔahrC* were evaluated in the polystyrene dish assay using TSB without dextrose and the heart valve assay. Error bars represent standard deviation. *indicates p<0.01 for less biofilm formation compared to OG1RF (for the same conditions). Note that in the heart valve assay the only strain with a significant reduction in biofilm formation compared to OG1RF is OG1RF *ΔahrC.*

In a previous study, OG1RF mutants with reduced biofilm formation in the polystyrene dish assay were assessed for virulence in an in vivo rabbit endocarditis model [15]. Mutants OG1RF Ω*argR*, OG1RF Ω*atlA*, and OG1RF Ω*pyrC* showed reduced biofilm formation in the polystyrene dish model but little or no attenuation of virulence in the rabbit endocarditis model. We reevaluated these same mutants using both the standard polystyrene dish assay and the ex vivo heart valve assay (Figure 4). As previously found, for biofilm formation in the polystyrene dish assay the strains were each attenuated compared to OG1RF (p<0.01 for all strains compared to OG1RF). However, for biofilm formation in the heart valve assay, none of the mutants differed significantly from OG1RF.

In the case of OG1RF *Δeep,* both the heart valve and polystyrene dish assays indicated that this mutant produced biofilm biomass and adherent bacterial CFU equivalent to wild type OG1RF (Figure 4), whereas its virulence was significantly attenuated in the rabbit endocarditis model [15], [24]. However, microscopic analysis of early biofilm development in this mutant revealed significant alterations in the cellular architecture of the biofilms [24], which may be relevant to virulence in the context of endocarditis. This suggests that both quantitative and qualitative aspects of biofilm development must be considered when assessing the role of biofilms in pathogenesis.

### Dextrose alters polystyrene dish assay results for some strains

Environmental conditions, such as the concentration of dextrose in the growth medium, have been shown to affect biofilm formation in the polystyrene dish assay [8]. The medium used for the heart valve assay contains 0.1% dextrose, while previous studies using the polystyrene dish assay have used various concentration of dextrose, including TSB without dextrose (0% dextrose) [15], TSB (0.25% dextrose) [20], or TSB with supplemental dextrose (>0.25%) [9]. Of note, these studies variously used the total biofilm biomass or the biofilm index (which normalizes for differences in planktonic growth), so the results are not necessarily directly comparable.

**Figure 5:**
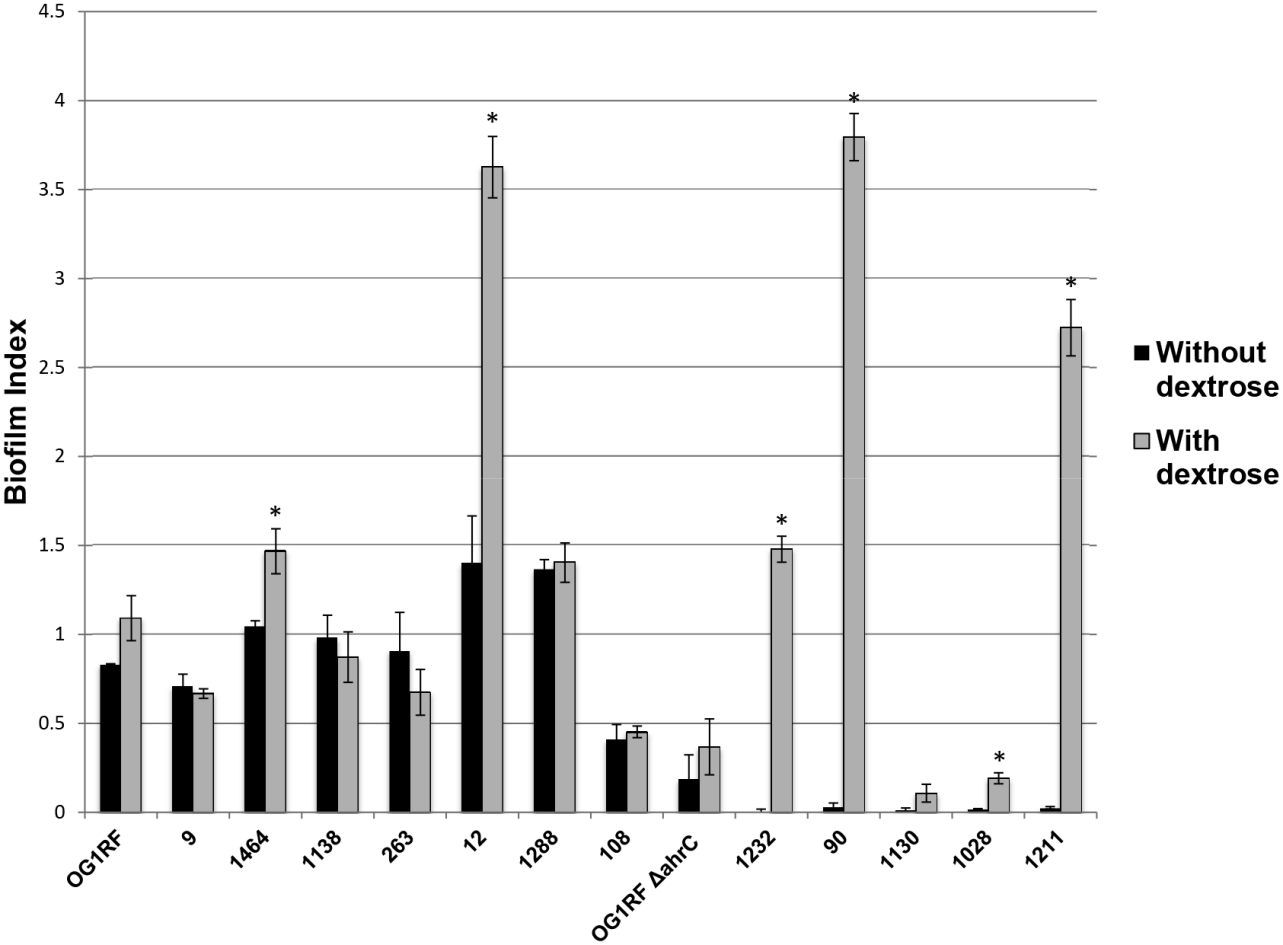
Biofilm index of clinical *Enterococcus faecalis* isolates grown in tryptic soy broth (TSB) with and without dextrose. Six isolates that showed high-level biofilm formation (biofilm index >1) (OG1RF, 9, 1464, 1138, 263, 12, and 1288), one with moderate biofilm formation (biofilm index 0.4) (180), and five that showed low-level biofilm formation (biofilm index <0.1) (OG1RF *ΔahrC*, 1232, 90, 1130, 1028, and 1211) in the standard assay (TSB without dextrose) were evaluated using TSB supplemented with dextrose (0.75% dextrose). Error bars represent standard deviation. *indicates p<0.01 for comparison of biofilm formation with and without dextrose.

To further investigate the potential role of dextrose in the differences noted between the polystyrene dish assay and the heart valve assay, 7 isolates that showed high-level biofilm formation (biofilm index >1), 1 isolate that showed moderate biofilm formation (biofilm index 0.4), and 6 isolates that showed low-level or no biofilm formation (biofilm index <0.1) in the standard polystyrene dish assay were evaluated for biofilm formation when grown using TSB with supplemental dextrose (0.75% dextrose) (Figure 5). High-level biofilm formers were OG1RF and clinical isolates 9, 1464, 1138, 263, 12, and 1288; the moderate biofilm former was isolate 108; and low-level biofilm formers were OG1RF *ΔahrC* and clinical isolates 1232, 90, 1130, 1028, and 1211. Of the 12 clinical isolates tested, only 6 (50%) had increased biofilm formation in the presence of 0.5% dextrose (p<0.01 for isolates 1232, 90, 1028, 1211, 1464, and 12 for comparison of biofilm formation with and without dextrose).

Of these 6 isolates with dextrose-dependent biofilm formation in the polystyrene tray assay, 5 (all except isolate 1028) are *esp*-positive (Table 1). Esp is known to play a role in dextrose-dependent biofilm formation in the polystyrene dish assay. Of special interest were the *esp*-positive isolates 12, 1464, 1138 and 1288. Isolates 12 and 1464 were high-level biofilm formers when grown without dextrose and had enhancement of biofilm formation in the presence of dextrose. In contrast, isolates 1138 and 1288 were high-level biofilm formers when grown without dextrose, but did not show enhancement with dextrose, even though they are also *esp-*positive.

**Table 1:** Characteristics of clinical *Enterococcus faecalis* study isolates.

Isolate^a^	Site of Isolation	*asa* b	*esp* c	Biofilm Index
1232	Urine	+	+	−0.03
90	Fecal	−	+	−0.02
1150	Urine	+	+	0.00
1037	Urine	+	+	0.00
1130	Urine	+	−	0.00
1028	Urine	+	−	0.00
99	Fecal	−	+	0.02
1211	Urine	−	+	0.02
103	Fecal	−	+	0.04
108	Fecal	−	−	0.43
1172	Urine	−	+	1.01
9	Fecal	−	−	1.05
1464	Wound	+	+	1.06
1138	Urine	+	+	1.09
263	Fecal	−	−	1.10
1278	Urine	+	−	1.32
1234	Urine	+	−	1.48
1288	Urine	+	+	1.60
12	Fecal	+	+	1.94

a *E. faecalis* isolates were collected through the Minneapolis Veteran’s Affairs Medical Center from 1994–1999.

b Aggregation substance gene.

c Enterococcal surface protein gene.

**Figure 6:**
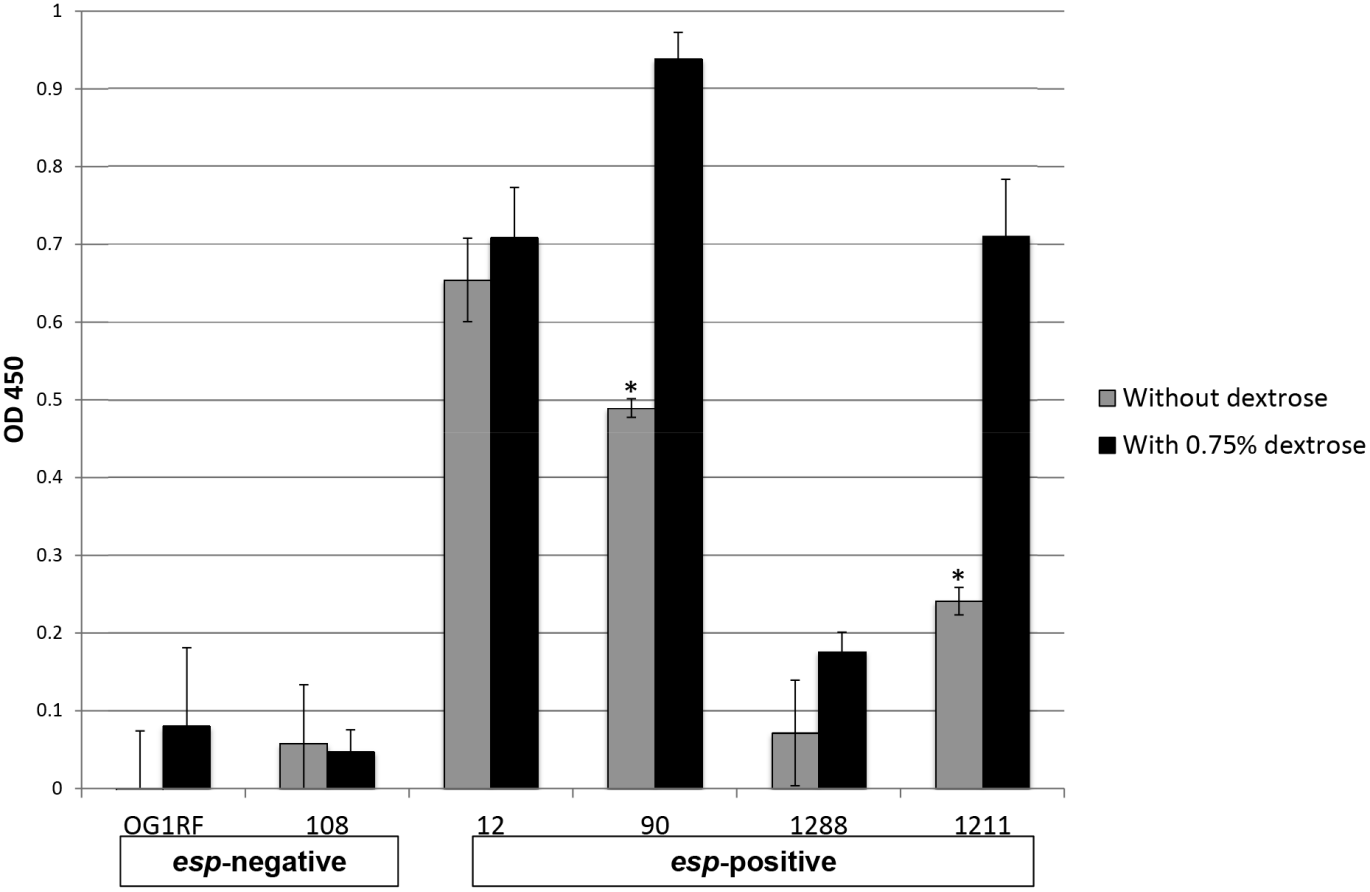
Expression of *esp* in tryptic soy broth (TSB) with and without 0.75% dextrose as assessed by enzyme linked immunosorbent assay (ELISA). *Enterococcus faecalis* isolates that were *esp*-negative (OG1RF and 108) or *esp* positive isolates (12, 90, 1288, and 1211) were assessed by ELISA for *esp* expression after growth in TSB without dextrose and TSB supplemented with dextrose (0.75% dextrose). The ELISA was repeated in duplicate with reproducible results. Some background signal, as seen in OG1RF and 108, is expected due to non-specific binding. Error bars represent standard deviation. *indicates p<0.05 for comparison of *esp* expression with and without dextrose.

To further explore the association between *esp*-positivity, dextrose supplementation, and biofilm formation, clinical *E. faecalis* isolates that are *esp-*negative (OG1RF and 108) or *esp*-positive (12, 90, 1288, and 1211) were grown in TSB with or without dextrose, then evaluated by ELISA for expression of *esp*. Isolates 90 and 1211, which showed the greatest dextrose-specific augmentation of biofilm formation, also showed the greatest dextrose-specific increase in *esp* expression (Figure 6).

## Discussion

In vitro biofilm assays using polystyrene dishes have served as the primary modality for evaluating the biofilm forming ability of bacterial isolates due to their ease of use and ability for high-throughput testing [8], [9], [10], [12]. Such assays are used to draw inferences about the role of biofilms in the pathogenesis of human infections and how best to treat biofilm-related infections [23], [25]. Several previous studies have evaluated biofilm formation using an ex vivo tissue model, as summarized in a recent review [26], but to our knowledge, no previous study has attempted to correlate biofilm formation in the polystyrene dish assay with biofilm formation on ex vivo tissue. However, two recently published studies have noted discordance between biofilm formation in the polystyrene dish assay and virulence in animal models [15], [27]. We show here that, for *E. faecalis*, results from the polystyrene dish biofilm assay correlate poorly with early biofilm formation on explanted heart valve tissue.

In contrast, the heart valve assay appears to correlate well with in vivo virulence in a rabbit endocarditis model for strain OG1RF *ΔahrC,* which exhibits a decrease in both ex vivo biofilm formation and in vivo virulence compared to wild-type. Because our cumulative results with the *Δeep* mutation suggest that both quantitative and qualitative aspects of biofilm development may affect virulence [15], [24], additional work is needed to assess whether this pattern will hold true with other mutations that attenuate biofilm formation and virulence.

There are many potential reasons for the poor correlation between the two biofilm assays, including differences in growth conditions, in the complexity and composition of the heart valve tissue surfaces vs. a polystyrene plate, and in the actual endpoints measured. Whereas the polystyrene dish assay measures biofilm biomass rather than number of CFU, the tissue assay involves enumeration of CFU. This might be expected to lead to discrepant assessments of biofilm formation, if at a given cell density some strains produce more extracellular matrix (i.e., biomass) than others. However, we found that CFU recovery from polystyrene dish biofilms matched very closely the corresponding biomass, as measured by safranin staining (Figure 1). This suggests that, at least for the isolates tested, the staining method used in the polystyrene dish assay closely approximates the number of cells present in the biofilm, and so does not explain the differences noted between assays.

Another methodological variable in the biofilm assays we studied was dextrose concentration, since the medium used for the heart valve assay has 0.1% dextrose, whereas the initial polystyrene dish assays were done in TSB with no dextrose. However, we repeated the polystyrene dish assay using TSB (0.25% dextrose) and found a similar pattern. The enterococcal surface protein gene *esp* has been shown to enhance biofilm formation in a dextrose-dependent fashion [9]. We documented dextrose-enhanced *esp* expression in some, but not all, *esp-*positive isolates, and those isolates also showed dramatic dextrose enhancement of biofilm formation on polystyrene. This suggests that changes in *esp* expression could explain some, but not all, of the differences noted between the polystyrene dish and heart valve assays. One hypothesis to explain the remainder of the difference is that enterococci have multiple surface adhesins that can mediate attachment to tissues. Our results suggest the possibility that only a subset of these, such as pili, function in the polystyrene dish assay.

We focused on the differences between biofilm formation on polystyrene vs. heart valve tissue surfaces, such as might be found in endocarditis. Future studies will be needed to determine whether the same patterns are true for other tissue-based infections such as wound infections. It remains unclear whether the polystyrene dish assay correlates well with in vivo biofilm formation on artificial materials such as catheters, although a study of enterococcal isolates causing catheter-related bloodstream infections suggested that polystyrene dish results did predict ability to cause biofilm-related infection [28]. These findings suggest that differing phenotypes in the polystyrene dish assay may be useful for predicting adhesion to some prosthetic devices. This would be a worthy area for future investigation.

Finally, we suggest that the ex vivo heart valve assay appears to have greater in vivo relevance for endocarditis than does the polystyrene dish assay, despite the latter’s convenience and high-throughput capability. Therefore, for studies using in vitro assays to assess the clinical relevance of enterococcal strains’ biofilm-forming ability, ex vivo biofilm formation on a relevant tissue surface should be considered either in primary screening, or as a means to validate results of in vitro assays.
